# Amino Acid Utilization May Explain Why *Bemisia tabaci* Q and B Differ in Their Performance on Plants Infected by the *Tomato yellow leaf curl virus*

**DOI:** 10.3389/fphys.2019.00489

**Published:** 2019-05-01

**Authors:** Litao Guo, Qi Su, Jin Yin, Zezhong Yang, Wen Xie, Shaoli Wang, Qingjun Wu, Hongying Cui, Youjun Zhang

**Affiliations:** ^1^Institute of Bast Fiber Crops, Chinese Academy of Agricultural Sciences, Changsha, China; ^2^Department of Plant Protection, Institute of Vegetables and Flowers, Chinese Academy of Agricultural Sciences, Beijing, China; ^3^Institute of Insect Sciences, College of Agriculture, Yangtze University, Jingzhou, China; ^4^Department of Entomology, College of Plant Protection, China Agricultural University, Beijing, China

**Keywords:** *Bemisia tabaci*, adaptation, *Tomato yellow leaf curl virus*, free amino acid, virus-herbivore interactions

## Abstract

To make plants more attractive to vectors of viruses, plant-infecting viruses can alter host plant physiology. The recent outbreaks of *Tomato yellow leaf curl virus* (TYLCV) relate to the spread of its primary vector, the whitefly *Bemisia tabaci*. Here, we investigated the question of whether the better performance of *B. tabaci* Q, relative to that of the B biotype, on TYLCV-infected tomato plants could be explained by differences in the ability of the *B. tabaci* Q and B to obtain free amino acids from the virus-infected plants. We found that the TYLCV infection of tomato plants significantly affected the mole percentage (mol%) of free amino acids in the phloem sap of the tomato plants and the mol% of free amino acids in *B. tabaci* adults and *B. tabaci* honeydew. The TYLCV infection caused the mol% of a larger number of free amino acids to rise in *B. tabaci* Q than in B, and the analysis of honeydew indicated that, when feeding on TYLCV-infected plants, *B. tabaci* Q was better able to use the free amino acids than *B. tabaci* B. The results suggest that *B. tabaci* Q is better adapted than B to feed on TYLCV-infected plants, and that TYLCV alters the *B. tabaci* B–Q competitive interaction in favor of Q.

## Introduction

*Bemisia tabaci* (Gennadius) (Hemiptera: Aleyrodidae) is a devastating agricultural pest worldwide (De Barro et al., 2011). It is a cryptic species complex consisting of at least 36 morphologically indistinguishable species (Boykin and De Barro, 2014) that differ in host range (Iida et al., 2009; Chu et al., 2012), feeding behavior (Liu et al., 2012), virus transmission (Pan et al., 2013a), insecticide resistance (Horowitz et al., 2005; Luo et al., 2010; Pan et al., 2015), or endosymbiont composition (Gottlieb et al., 2006; Chiel et al., 2007). Two of the most invasive and devastating genotypes of the species are B (Middle East-Asia Minor 1) and Q (Mediterranean) (Dinsdale et al., 2010; De Barro et al., 2011). In most parts of China, *B. tabaci* Q has gradually displaced *B. tabaci* B and has become the predominant *B. tabaci* genotype (Pan et al., 2011, 2015; Zheng et al., 2017).

Because of their polyphagous nature and adaptability, *B. tabaci* B and Q are highly invasive (Inbar and Gerling, 2008). *B. tabaci* B and Q have spread in as many as 60 countries during the last two decades (De Barro et al., 2011; Pan et al., 2015). *B. tabaci* harm plants by transmitting 311 plant viruses, sucking phloem sap, and excreting honeydew (Gilbertson et al., 2015). The rapid spread of *B. tabaci* B and Q has come together with outbreaks of begomoviruses in the cropping systems of China and many other countries (Shi et al., 2014).

As a single-stranded-DNA plant virus, *Tomato yellow leaf curl virus* (TYLCV) is phloem-limited, exhibits tissue tropism in the plant phloem, and produces characteristic symptoms on plants (Czosnek and Ghanim, 2002). In many tropical and subtropical areas, it is a destructive pathogen of the Solanaceae and causes significant yield losses. Within *B. tabaci* populations, TYLCV is transmitted transovarially, i.e., from female whiteflies to offspring, contributing significantly to its global spread (Ghanim et al., 1998; Wei et al., 2017). When feeding on a TYLCV-infected host plant, *B. tabaci* ingests TYLCV virions through the stylet. The ingested virions are then delivered to midgut epithelial cells, from where they moved to the hemolymph, and circulate until they access the salivary glands, which enables transmission to the plant phloem (Cicero et al., 1995; Hunter et al., 1998; Ghanim et al., 2001; Czosnek and Ghanim, 2002).

Plants are frequently damaged by insects and insect-vectored pathogens. In plant–pathogen–vector systems, the pathogen can directly affect the insect vector or indirectly affect the insect vector through an alteration of plant physiology (Belliure et al., 2005; Colvin et al., 2006; Stout et al., 2006). For example, Stout et al. (2006) studied nutrition-related interactions between aphids and virus-infected plants and concluded that the performance of aphids is often related to the nutritional quality of phloem sap as phloem-feeders, aphids, and other phloem feeding insects, such as whiteflies, absorb a diet that contains fairly high levels of free amino acids (Buchanan et al., 2000). Host selection and insect development are correlated with the diet’s relative quality and feeding efficiency (Montllor, 1989). Many studies have examined how such tripartite interactions affect the population dynamics of insect vectors and plant pathogens, and the invasiveness of alien species (Colvin et al., 2006; Stout et al., 2006; Jiu et al., 2007; Pan et al., 2013a; Su et al., 2015, 2016).

Recent research has indicated that TYLCV-infected host plants have different effects on *B. tabaci* B and Q host preference and feeding behavior (Fang et al., 2013; Liu et al., 2013). In general, TYLCV and *B. tabaci* B seem to be neutral or antagonistic (Liu et al., 2009; Pan et al., 2013a; Shi et al., 2013), whereas TYLCV and *B. tabaci* Q seem to be mutualistic or neutral (Matsuura and Hoshino, 2009; Li et al., 2011; Pan et al., 2013a; Shi et al., 2013). However, the mechanisms underlying the nutrition-related interactions (especially with respect to free amino acids) are not completely understood.

For the present research, we hypothesized that *B. tabaci* Q was better adapted than B for feeding on TYLCV-infected tomato plants and that this difference was associated with variations in the levels of free amino acids in the plants, the whiteflies, and the honeydew produced by these whiteflies. We first examined how the free amino acid composition of the phloem sap of tomato plants was modified by TYLCV infection. After allowing B and Q adults to feed on healthy and virus-infected tomato plants, we assessed how virus-induced changes in phloem amino acids affect the nutritional status of *B. tabaci* B and Q, as indicated by the composition of free amino acids in the adults and in their honeydew.

## Materials and Methods

### Plant Cultures and *B. tabaci* Populations

Tomato (*Solanum lycopersicum* Miller, cv. Zhongza 9) was used in our experiments and were held in a glasshouse with natural light and a controlled temperature (26 ± 2°C).

*Bemisia tabaci* Q specimens were collected from poinsettia, *Euphorbia pulcherrima* Wild. EX Klotz., in Beijing, China in 2009, whereas *B. tabaci* B whiteflies were originally collected in 2004 from a cabbage field, *Brassica oleracea* L. cv. Jingfeng 1, in Beijing, China (Pan et al., 2012).

From the time of their collection, the *B. tabaci* B and Q insects used in this study were reared on tomato (*S. lycopersicum* Mill. cv. Zhongza 9), in a glasshouse with natural light and a controlled temperature (26 ± 2°C). The method used for monitoring the purity of the populations was the same as that described previously (Chu et al., 2010).

### TYLCV Inoculation

In our experiments, the method of TYLCV inoculation was the same as that described previously (Pan et al., 2013a). The GenBank accession ID of the TYLCV genome is AM282874.

### Amino Acid Analyses

#### Sampling and Assaying of Amino Acids in the Phloem Sap

To assess the impact of the TYLCV infection on plant nutritional quality, we collected and analyzed the phloem sap of healthy and TYLCV-infected tomato plants as described by Su et al. (2015). In brief, phloem sap from the fifth expanded leaf was sampled. The leaf was immersed in 600 μl of 5 mM Na_2_EDTA (pH 7.5). The leaf in the EDTA solution was incubated in a light-proof box at 25°C; a saturated solution of KH_2_PO_4_ was put in the box to maintain high relative humidity. After 90 min, the leaf was discarded and the phloem exudate in the EDTA solution was frozen at -20°C until it was used for amino acid analysis. Free amino acid content of phloem exudates were analyzed with an automatic amino acid analyzer S433 (Sykam, Munich, Germany). All analyses were performed on three biological replicates.

#### Sampling and Assaying of Amino Acids in *B. tabaci* Adults

Newly emerged B and Q adults were collected from healthy and TYLCV-infected tomato plants. We analyzed the amino acid content of B. tabaci adults using the approach described in Pan et al. (2013b). A 20 mg whitefly adult (representing one replicate) was fully homogenized with a 2 mL-glass homogenizer, shaken for 2 min on the vortex shaker (QL-866, Qilinbeier), and then centrifuged at 14,000 rpm for 10 min in centrifuge (5417R, Eppendorf, Germany). One mL of the supernatant was mixed with an equal volume of n-hexane. The mixture was then centrifuged at 10,000 rpm for 10 min, after which the supernatant was discarded and 0.5 mL of the underlayer was drawn and mixed with an equal volume of 8% sulfosalicylic acid. The latter was centrifuged at 10,000 rpm for 10 min (to remove protein). Then, 0.5 mL of the supernatant was concentrated to dryness and re-dissolved in 0.75 mL of double-distilled water. The extracts were passed through a 0.45 μm filter, and an analysis of free amino acid content was performed as described for phloem sap. All analyses were performed on three biological replicates.

#### Sampling and Assaying of Amino Acids in the Honeydew of *B. tabaci* Adults

Newly emerged B and Q adults feeding on healthy tomato plants were moved to the TYLCV-infected or healthy tomato plants. These whiteflies were placed on the back side of leaves (50 adults per leaf), and their honeydew was collected on aluminum foil in a clip collection cage (2.5 cm diameter) for 48 h (Wilkinson and Douglas, 1995). The honeydew should be kept dry because the amino acids of honeydew would be broken during collection (Sandström and Moran, 2001), and was dried in a Speed-vac. The dry honeydew samples were dissolved in 50 μL of 80% methanol, and an analysis of free amino acid content was performed as described for phloem sap. All analyses were performed on three biological replicates.

### Statistical Analyses

The concentration of every amino acid was transformed to the mole percentage (mol%) of total amino acids. A one-way analysis of the variance (ANOVA) and the least significant difference (LSD) test (SPSS 17.0 for Windows; SPSS, Chicago, IL, United States) were used to compare the mol% of individual amino acids in the phloem sap of healthy and TYLCV-infected tomato plants. A two-way analysis of the variance and the LSD test (SPSS 17.0 for Windows; SPSS, Chicago, IL, United States) was used to compare the mol% of individual amino acids of whiteflies whole body and honeydew.

## Results

### Free Amino Acids in the Phloem Sap of Healthy and TYLCV-Infected Tomato Plants

**FIGURE 1:**
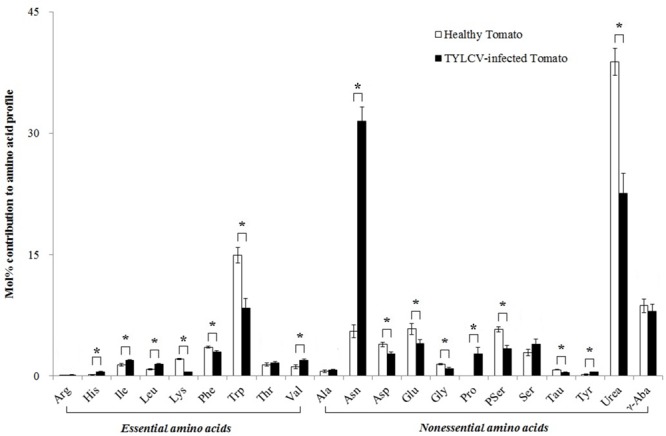
The mol% of free amino acids in the phloem sap of healthy and TYLCV-infected tomato plants. Values are means (± SE) of three replicates. ^∗^Indicates significant differences between healthy and TYLCV-infected tomato plants at *P* < 0.05; LSD test. For abbreviations, see Table 1.

Twenty free amino acids were detected in healthy tomato plants, and the same 20 plus proline (Pro) were detected in TYLCV-infected tomato plants. The TYLCV infection increased the mol% of histidine (His) (+212%, *F*_1,8_ = 92.159, *P*< 0.001), isoleucine (Ile) (+42%, *F*_1,8_ = 6.937, *P*= 0.030), leucine (Leu) (+79%, *F*_1,8_ = 13.769, *P*= 0.006), valine (Val) (+70%, *F*_1,8_ = 7.468, *P*= 0.026), asparagine (Asn) (+470%, *F*_1,8_ = 181.163, *P*< 0.001), and tyrosine (Tyr) (+153%, *F*_1,8_ = 11.007, *P*= 0.011) in the phloem sap of tomato plants. However, the virus infection decreased the relative concentration of lysine (Lys) (-77%, *F*_1,8_ = 92.159, *P*< 0.001), phenylalanine (Phe) (-16%, *F*_1,8_ = 8.384, *P*= 0.020), tryptophan (Trp) (-43%, *F*_1,8_ = 19.116, *P*= 0.002), aspartate (Asp) (-29%, *F*_1,8_ = 10.538, *P*= 0.012), glutamate (Glu) (-30%, *F*_1,8_ = 6.011, *P*= 0.040), glycine (Gly) (-37%, *F*_1,8_ = 17.374, *P*= 0.003), phosphoserine (PSer) (-41%, *F*_1,8_ = 20.778, *P*= 0.002), taurine (Tau) (-41%, *F*_1,8_ = 18.516, *P*= 0.003), and urease (Urea) (-42%, *F*_1,8_ = 29.528, *P*= 0.001) in the phloem sap of tomato plants. (Figure 1).

### Free Amino Acids in *B. tabaci* B and Q Adults

A total of 24 free amino acids were detected in both *B. tabaci* B and Q adults that fed on infected and healthy plants (Table 1). Genotypes significantly affected the mol% of the essential amino acids arginine (Arg), Ile, and Phe in the adults (Table 1). Genotypes also significantly affected the mol% of the non-essential amino acids ornithine (Orn) and β-aminoisobutyric acid (β-AiBA) in the adults. Virus infection significantly influenced the mol% of the essential amino acids Arg and Trp, and of the non-essential amino acids Asn, cysteine (Cys), Glu, Gly, Orn, Pro, Tyr, and β-AiBA in adults. The interaction of genotypes and the virus infection significantly affected the mol% of the essential amino acid Phe in adults (Table 1).

**Table 1:** ANOVA results for the effects of *B. tabaci* genotypes and virus (TYLCV) on the mol% of free amino acids in *B. tabaci* adults.

Amino acid*^a^*		Genotype*^c^*	Virus*^d^*	Genotype^∗^virus
	Arginine (Arg)	^∗^	^∗∗^	n.s.
	Histidine (His)	n.s.	n.s.	n.s.
	Isoleucine (Ile)	^∗^	n.s.	n.s.
	Leucine (Leu)	n.s.	n.s.	n.s.
Essential	Lysine (Lys)	n.s.	n.s.	n.s.
amino acids*^b^*	Methionine (Met)	n.s.	n.s.	n.s.
	Phenylalanine (Phe)	^∗^	n.s.	^∗^
	Threonine (Thr)	n.s.	n.s.	n.s.
	Tryptophan (Trp)	n.s.	^∗∗^	n.s.
	Valine (Val)	n.s.	n.s.	n.s.
	Alanine (Ala)	n.s.	n.s.	n.s.
	Asparagine (Asn)	n.s.	^∗∗^	n.s.
	Aspartate (Asp)	n.s.	n.s.	n.s.
	Cysteine (Cys)	n.s.	^∗^	n.s.
	Glutamate (Glu)	n.s.	^∗∗^	n.s.
	Glycine (Gly)	n.s.	^∗^	n.s.
	Ornithine (Orn)	^∗∗∗^	^∗∗^	n.s.
Non-essential	Proline (Pro)	n.s.	^∗∗∗^	n.s.
amino acids	Serine (Ser)	n.s.	n.s.	n.s.
	Tyrosine (Tyr)	n.s.	^∗∗^	n.s.
	α-Aminoadipic acid (α-Aaa)	n.s.	n.s.	n.s.
	β-Alanine (β-Ala)	n.s.	n.s.	n.s.
	β-Aminoisobutyric acid (β-AiBA)	^∗^	^∗^	n.s.
	γ-Aminobutyric acid (γ-Aba)	n.s.	n.s.	n.s.

*^*a*^Abbreviated amino acid names are in parentheses. ^*b*^Essential amino acids as defined by Morris (1991). ^*c*^B. tabaci B and B. tabaci Q. ^*d*^Tomato plants with and without TYLCV. ^∗, ∗∗^, and ^∗∗∗^ indicate *P* < 0.05, *P* < 0.01, and *P* < 0.001 (LSD test, n = 3), respectively; n.s. indicates non-significant.*

**FIGURE 2:**
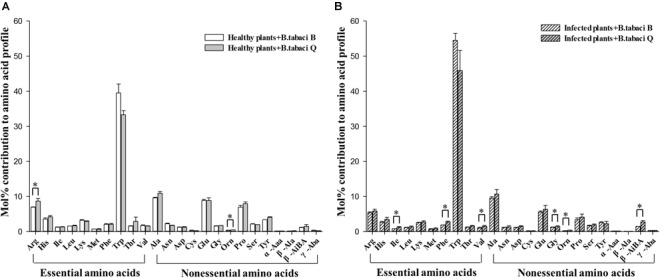
The mol% of free amino acids in *B. tabaci* B and Q adults that fed on **(A)** healthy tomato plants or on **(B)** TYLCV-infected tomato plants. Values are means (± SE) of three replicates. ^∗^Indicates a significant difference between B and Q at *P* < 0.05; LSD test. For abbreviations, see Table 1.

When adults fed on healthy plants, the mol% of Arg and Orn were higher in Q than in B (Figure 2A and Supplementary Table S1); Arg is essential, but Orn is not. When adults fed on virus-infected plants, the mol% of Ile, Phe, Val, Gly, Orn, and β-AiBA were higher in Q than in B (Figure 2B and Supplementary Table S1); Ile, Phe, and Val are essential amino acids, but the others are not. When adults fed on either TYLCV-infected or healthy tomato plants, the mol% was not significantly higher in B than in Q for any free amino acid (Figure 2).

### Free Amino Acids in the Honeydew of *B. tabaci* Adults

A total of 22 free amino acids were found in the honeydew of *B. tabaci* adults that fed on TYLCV-infected and healthy tomato plants. Genotypes significantly influenced the mol% of the essential amino acids Ile, threonine (Thr), and Val, and the mol% of the non-essential amino acids Glu, Pro, Tyr, and β-Alanine (β-Ala) in the honeydew. The virus infection significantly affected the mol% of all the essential amino acids except Arg, and significantly affected the mol% of the non-essential amino acids Asn, Asp, Glu, Orn, Pro, and Tyr in the honeydew. The interaction of genotypes and the virus significantly affected the mol% of the essential amino acid Ile and of the non-essential amino acid β-Ala in the honeydew (Table 2).

**Table 2:** ANOVA results for the effects of *B. tabaci* genotypes and virus (TYLCV) on the mol% of free amino acids in the honeydew of *B. tabaci* adults.

Amino acid*^a^*		Genotypes*^c^*	Virus*^d^*	Genotype^∗^virus
	Arg	n.s.	n.s.	n.s.
	Ile	^∗^	^∗∗∗^	^∗^
	Leu	n.s.	^∗^	n.s.
Essential	Lys	n.s.	^∗∗^	n.s.
amino acids*^b^*	Met	n.s.	^∗∗^	n.s.
	Phe	n.s.	^∗^	n.s.
	Thr	^∗∗^	^∗∗^	n.s.
	Trp	n.s.	^∗∗^	n.s.
	Val	^∗^	^∗∗^	n.s.
	Ala	n.s.	n.s.	n.s.
	Asn	n.s.	^∗∗^	n.s.
	Asp	n.s.	^∗∗^	n.s.
	Glu	^∗∗^	^∗∗^	n.s.
	Gly	n.s.	n.s.	n.s.
	Orn	n.s.	^∗∗∗^	n.s.
Non-essential	Pro	^∗^	^∗∗^	n.s.
amino acids	Ser	n.s.	n.s.	n.s.
	Tyr	^∗^	^∗∗^	n.s.
	α-Aaa	n.s.	n.s.	n.s.
	β-Ala	^∗∗^	n.s.	^∗∗^
	β-AiBA	n.s.	n.s.	n.s.
	γ-Aba	n.s.	n.s.	n.s.

*^*a*^Abbreviated names of amino acid. ^*b*^Essential amino acids as defined by Morris (1991). ^*c*^B. tabaciB and B. tabaci Q. ^*d*^Tomato plants with and without TYLCV. ^∗, ∗∗^, and ^∗∗∗^ indicate *P* < 0.05, *P* < 0.01, and *P* < 0.001 (LSD test, n = 3), respectively; n.s. indicates non-significant.*

**FIGURE 3:**
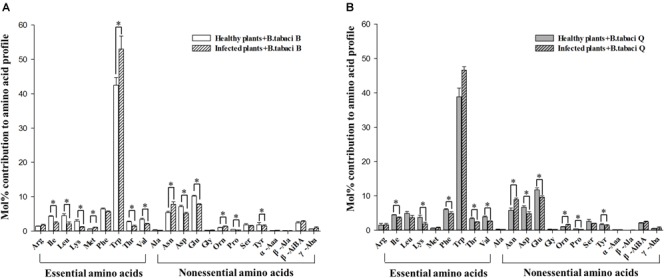
The mol% of free amino acids in the honeydew of **(A)** *B. tabaci* B adults and **(B)** *B. tabaci* Q adults that fed on healthy and TYLCV-infected tomato plants. Value are means (± SE) of three replicates. ^∗^Indicates a significant difference between healthy and infected plants at *P* < 0.05; LSD test. For abbreviations, see Table 1.

The mol% of five essential amino acids (Ile, Leu, Lys, Thr, and Val) and four non-essential amino acids (Asp, Glu, Pro, and Tyr) were lower in the honeydew produced by B adults that fed on TYLCV-infected tomato plants rather than on healthy plants, and the mol% of two essential amino acid (Met and Trp) and two non-essential amino acids (Asn and Orn) were higher in the honeydew produced by B adults that fed on TYLCV-infected tomato plants rather than on healthy plants (Figure 3A, Table 3 and Supplementary Table S2). The mol% of five essential amino acids (Ile, Lys, Phe, Thr, and Val) and four non-essential amino acids (Asp, Glu, Pro, and Tyr) were lower in the honeydew produced by Q adults that fed on TYLCV-infected tomato plants than on healthy plants, and the mol% of only two non-essential amino acids (Asn and Orn) were higher in the honeydew produced by Q adults that fed on TYLCV-infected tomato plants rather than on healthy plants (Figure 3B, Table 3 and Supplementary Table S2).

**Table 3:** The number of free amino acids whose mol% were higher or lower in the honeydew of *B. tabaci* adults (genotypes B and Q) that fed on TYLCV-infected tomato plants vs. healthy tomato plants.

Mol%^a^	Genotypes	Free amino acids in the honeydew	
		**Essential amino acids**	**Non-essential amino acids**
Lower	B	5 (Ile, Leu, Lys, Thr, Val)	4 (Asp, Glu, Pro, Tyr)
	Q	5 (Ile, Lys, Phe, Thr, Val)	4 (Asp, Glu, Pro, Tyr)
Higher	B	2 (Met, Trp)	2 (Asn,Orn)
	Q	0	2 (Asn, Orn)

*^*a*^Lower and Higher indicate that the mol% were lower or higher, respectively, in the honeydew derived from virus-infected plants than in the honeydew derived from healthy plants.*

## Discussion

Research has shown that vectored viruses can alter host plant phenotypes so as to change interactions with other organisms, including interactions between plants, viruses, and insect vectors of viruses (Mauck et al., 2012, 2018; Casteel and Falk, 2016; Eigenbrode and Bosque-Perez, 2016; Mauck, 2016). Insect-vectored viruses can alter many host plant factors, including odors, induced defenses, visual and tactile characteristics, sugars, free amino acids, and secondary metabolites (Bosque-Perez and Eigenbrode, 2011; Casteel et al., 2014; Mauck et al., 2014a,b). In our study, TYLCV significantly altered the free amino acid concentration in the phloem sap of tomato plants (Figure 1), an observation that is consistent with earlier studies on other interactions between plants and pathogens (Casteel et al., 2014; Su et al., 2015). Amino acids are important nutrients because they are required for cell growth regulation, hormone metabolism, nerve transmission, protein synthesis, the production of metabolic energy, and nitrogen metabolism (Castagna et al., 1997; Curis et al., 2007; Manna et al., 2009; Wu, 2009; Wu et al., 2014). Lys and Asn are directly related to antiviral activity and the regulation of the immune function, respectively, and Trp is the only amino acid with enhanced immune function (Wu, 2009). Posttranslational modifications of Lys are related to *Leishmania* survival (Nayak et al., 2018). In an earlier study, a positive correlation was observed between the number of *B. tabaci* individuals (feeding and eggs) and the amino acid content of a plant (Crafts-Brandner, 2002). The concentrations of Ser, Ala, Pro, Phe, Asn, Glu, Asp, Arg, and Trp play a role in the survival rate of *B. tabaci*, while the concentrations of Asp, Glu, Arg, His, and Asn are related to oviposition by *B. tabaci* (Thompson, 2006). In our study, the mol% of Lys, Trp, and Urea were much lower, and the mol% of Asn was much higher in TYLCV-infected tomato plants than in healthy tomato plants. Our results are consistent with a previous study that found that TYLCV increases free amino acids (His, Ile, Leu, Val, Asn, and Tyr) in the infected tomato phloem sap (Su et al., 2015); the latter study also found that TYLCV attenuates the induction of defenses against *B. tabaci* Q. In contrast to the latter study, the current research assessed the effects of TYLCV on both *B. tabaci* Q and B to advance our understanding of how the virus might affect competition between the two genotypes.

Because virus-infected plants often display better nutritional quality, more efficient absorption of nutrients, or repressed anti-herbivore defenses, many insect herbivores select virus-infected plants (Mauck et al., 2012; Wang et al., 2012; Angeles-López et al., 2016). Our study revealed that *B. tabaci* Q is better able to use TYLCV-infected plants as a source of amino acids, as compared with *B. tabaci* B. In our study, when feeding on TYLCV-infected tomato plants, *B. tabaci* Q had a higher mol% of amino acids (Ile, Phe, Val, Gly, Orn, and β-AiBA) than *B. tabaci* B (Figure 2B and Supplementary Table S1). TYLCV also had different effects on the mol% of some free amino acids in *B. tabaci* Q and B adults. The amino acid mol% of *B. tabaci* Q was relatively high as a consequence of feeding on TYLCV-infected plants, indicating that *B. tabaci* Q is better adapted to feeding on TYLCV-infected tomato plants than *B. tabaci* B. As is well known, many amino acids, especially essential amino acids obtained through the diet, cannot be synthesized in insects, but are necessary for normal development (Hansen and Moran, 2011; Boudko, 2012). Research on aphid-virus-host interactions has shown that aphid performance is associated with the nutritional quality of phloem sap (Stout et al., 2006). For example, *Aphis gossypii* Glover feeding on *Zucchini yellow mosaic virus*-infected *Cucurbita pepo* had longer longevity and higher fecundity than when feeding on healthy plants. In addition, the differences were associated with higher amino acid concentrations in the virus-infected plant’s phloem sap (Blua et al., 1994). In contrast, lowered concentrations of amino acids in the phloem sap of wheat plants infected by two *Barley yellow dwarf virus* strains reduced the suitability of wheat for the aphid *Sitobion avenae* (Fabricius) (Fiebig et al., 2004). In the latter study, the assimilation of amino acids was also lower for aphids feeding on virus-infected plants than on non-infected plants (Fiebig et al., 2004).

We analyzed amino acids in honeydew excreted by *B. tabaci* adults to assess the assimilation of amino acids by adults. The number of free amino acids whose mol% in honeydew was reduced by the TYLCV infection of tomato plants was the same for *B. tabaci* Q and B, but the number of free amino acids whose mol% in honeydew was increased by TYLCV infection was less for *B. tabaci* Q than B (Table 3). This result suggests that the efficiency of amino acid utilization was higher in *B. tabaci* Q than for B. The changes in the mol% of free amino acids in honeydew may help explain why *B. tabaci* Q performs better than *B. tabaci* B on TYLCV-infected plants (Pan et al., 2013a).

Taken together, our study shows that *B. tabaci* Q is better adapted than *B. tabaci* B for feeding on TYLCV-infected tomato plants. These results are in agreement with earlier observations indicating that *B. tabaci* Q may more effectively spread TYLCV than *B. tabaci* B, and that *B. tabaci* Q performs better on TYLCV-infected plants than on healthy plants (Pan et al., 2013a). This mutualistic relationship between TYLCV and *B. tabaci* Q may help explain why *B. tabaci* Q has gradually displaced *B. tabaci* B during TYLCV outbreaks in China and elsewhere.

## Author Contributions

LG, JY, and YZ conceived and designed the experiments. LG and JY performed the experiments. LG and QS analyzed the data. LG, SW, QW, ZY, WX, HC, and YZ contributed to the reagents, materials, and analysis tools. LG, QS, JY, WX, SW QW, and YZ wrote the manuscript.

## Conflict of Interest Statement

The authors declare that the research was conducted in the absence of any commercial or financial relationships that could be construed as a potential conflict of interest.
