# Supplementary material for: Amino Acid Utilization May Explain Why Bemisia tabaci Q and B Differ in Their Performance on Plants Infected by the Tomato yellow leaf curl virus
Source: Front Physiol. 2019 May 1;10:489. doi: 10.3389/fphys.2019.00489 (PMC6504830; doi:10.3389/fphys.2019.00489)
Supplement: Supplementary file 1 [file Data_Sheet_1.docx]

**Table S1**. **The mol% of individual amino acids in *B. tabaci* B and Q adults that fed on healthy or TYLCV-infected tomato plants.**

| **Amino acid** | **Healthy plants+ B** | **Healthy plants+ Q** | **Infected plants+ B** | **Infected plants+ Q** |
| --- | --- | --- | --- | --- |
| Arg | 6.91±0.08 b | 8.64±0.74 a | 5.40±0.20 b | 5.84±0.53 b |
| His | 3.52±0.31 ab | 4.21±0.34 a | 2.65±0.25 b | 3.45±0.55 ab |
| Ile | 1.17±0.13 a | 1.30±0.05 a | 0.77±0.03 b | 1.25±0.16 a |
| Leu | 1.51±0.06 a | 1.61±0.17 a | 1.13±0.10 a | 1.33±0.23 a |
| Lys | 3.23±0.17 a | 2.88±0.12 ab | 2.53±0.08 b | 2.70±0.32 ab |
| Met | 0.69±0.06 a | 0.60±0.18 a | 0.64±0.14 a | 0.78±0.23 a |
| Phe | 2.06±0.14 b | 2.05±0.27 b | 1.86±0.02 b | 2.74±0.22 a |
| Trp | 39.53±2.56 bc | 33.28±1.18 c | 54.50±2.01 a | 45.92±5.72 ab |
| Thr | 1.55±0.04 a | 2.90±1.21 a | 1.20 ±0.08 a | 1.56±0.17 a |
| Val | 1.70±0.15 a | 1.56±0.05 a | 0.98 ±0.19 b | 1.43±0.31 a |
| Ala | 9.62±0.15 a | 10.92±0.47 a | 9.49 ±0.46 a | 10.64±1.30 a |
| Asn | 2.21±0.16 a | 1.74±0.02 ab | 1.16 ±0.07 c | 1.33±0.29 bc |
| Asp | 1.24±0.07 a | 1.25±0.15 a | 1.15 ±0.07 a | 1.50±0.16 a |
| Cys | 0.31±0.05 a | 0.21±0.02 ab | 0.15 ±0.05 b | 0.13±0.05 b |
| Glu | 8.82±0.38 a | 8.87±0.72 a | 5.51 ±0.35 b | 6.36±1.08 b |
| Gly | 1.58±0.06 a | 1.68±0.01 a | 1.23±0.06 b | 1.38±0.24 a |
| Orn | 0.31±0.03 b | 0.45±0.01 a | 0.21±0.01 c | 0.37±0.03 b |
| Pro | 6.90±0.49 a | 7.93±0.46 a | 3.33±0.50 b | 4.09±0.88 b |
| Ser | 2.12±0.11 a | 1.96±0.03 a | 1.63 ±0.11 a | 1.83±0.26 a |
| Tyr | 3.34±0.05 ab | 4.00±0.23 a | 2.54±0.26 b | 2.18±0.69 b |
| α-Aaa | 0.12±0.02 a | 0.11±0.02 a | 0.16±0.00 a | 0.14±0.02 a |
| β-Ala | 0.11±0.01 a | 0.13±0.08 a | 0.08±0.01 a | 0.04±0.02 a |
| β-AiBA | 1.15±0.03 b | 1.50±0.45 b | 1.43±0.04 b | 2.73±0.35 a |
| γ-Aba | 0.30±0.06 a | 0.23±0.01 a | 0.27±0.02 a | 0.26±0.02 a |

The mol% of amino acids are the means (± SE) of three biological replicates. Lowercase indicates a significant difference among four experiments (LSD test, P < 0.05). For abbreviations, see Table 1.

**Table S2**. **The mol% of individual amino acids in the honeydew excreted by *B. tabaci* B and Q adults that fed on healthy or TYLCV-infected tomato plants.**

| **Amino acid** | **Healthy plants+ B** | **Healthy plants+ Q** | **Infected plants+ B** | **Infected plants+ Q** |
| --- | --- | --- | --- | --- |
| Arg | 1.38±0.07 a | 1.50±0.45 a | 1.70±0.28 a | 1.55±0.37 a |
| Ile | 4.28±0.18 ab | 4.47±0.09 a | 2.38±0.32 d | 3.62±0.24 bc |
| Leu | 4.42±0.64 a | 4.87±0.55 a | 2.08±0.57 b | 3.62±0.62 ab |
| Lys | 2.87±0.43 ab | 3.72±0.65 a | 1.09±0.24 c | 1.82±0.47 bc |
| Met | 0.58±0.09 b | 0.57±0.11 b | 1.08±0.05 a | 0.75±0.12 b |
| Phe | 6.46±0.26 a | 6.01±0.24 a | 5.62±0.22 ab | 4.88±0.45 b |
| Trp | 42.46±2.27 b | 38.81±2.61 b | 53.03±3.76 a | 46.65±0.99 ab |
| Thr | 2.66±0.21 ab | 3.37±0.23 a | 1.42±0.32 d | 2.41±0.06 bc |
| Val | 3.34±0.39 ab | 3.89±0.24 a | 2.05±0.16 c | 2.65±0.01 bc |
| Ala | 0.33±0.06 a | 0.25±0.06 a | 0.22±0.01 a | 0.23±0.02 a |
| Asn | 5.40±0.35 b | 5.76±0.68 b | 7.76±0.73 a | 8.88±0.44 a |
| Asp | 7.11±0.37 a | 6.71±0.41 a | 5.09±0.37 b | 4.79±0.56 b |
| Glu | 10.19±0.26 b | 11.69±0.63 a | 7.75±0.20 c | 9.55±0.43 b |
| Gly | 0.20±0.02 a | 0.21±0.05 a | 0.22±0.06 a | 0.19±0.02 a |
| Orn | 0.99±0.05 b | 0.99±0.08 b | 1.36±0.09 a | 1.61±0.07 a |
| Pro | 0.43±0.00 a | 0.37±0.02 ab | 0.29±0.04 bc | 0.21±0.03 c |
| Ser | 1.80±0.32 ab | 2.55±0.55 a | 1.45±0.18 b | 1.94±0.12 ab |
| Tyr | 1.80±0.64 a | 1.60±0.31 ab | 1.46±0.29 bc | 1.33±0.31 c |
| α-Aaa | 0.15±0.02 ab | 0.13±0.01 b | 0.18±0.01 a | 0.15±0.00 ab |
| β-Ala | 0.15±0.02 a | 0.01±0.00 a | 0.07±0.03 a | 0.08±0.01 a |
| β-AiBA | 2.40±0.28 ab | 2.06±0.12 b | 2.79±0.27 a | 2.46±0.19 ab |
| γ-Aba | 0.59±0.08 a | 0.48±0.04 a | 0.89±0.31 a | 0.64±0.34 a |

The mol% of amino acids are the means (± SE) of three biological replicates. Lowercase indicates a significant difference among four experiments (LSD test, P < 0.05). For abbreviations, see Table 1.
